# Eating Behaviors in Cuban Adults: Results from an Exploratory Transcultural Study

**DOI:** 10.3389/fpsyg.2016.01455

**Published:** 2016-09-26

**Authors:** Boris C. Rodríguez-Martín, Marco Innamorati, Claudio Imperatori, Mariantonietta Fabbricatore, Désirée Harnic, Luigi Janiri, Saira R. Rivas-Suárez

**Affiliations:** ^1^Department of Psychology, Central University “Marta Abreu” of Las VillasSanta Clara, Cuba; ^2^Department of Human Sciences, Università Europea di RomaRome, Italy; ^3^Bipolar Disorders Unit, Psychiatric Day Hospital, Catholic University of Sacred HeartRome, Italy; ^4^Policlinico Universitario Agostino GemelliRome, Italy; ^5^Department of Morphophysiology, Medical University of Villa Clara “Serafín Ruiz de Zárate Ruiz”Santa Clara, Cuba

**Keywords:** eating behaviors, food thought suppression, food cravings, binge eating, motivation

## Abstract

This study aims to investigate eating behaviors in Cuban adults and compare them with those of a developed Western country, Italy. The study also aimed to determine the overall accuracy of a predictive model intended to define variables which could be used to discriminate between nationalities. Participants were 283 normal weight individuals from Cuba (*n* = 158) and Italy (*n* = 125). Italians had higher scores for restrained eating on the questionnaire than Cubans with a considerable effect size. This trend was also found for emotional eating and binge eating, as well as number of current dieters, despite the fact that effect sizes were small. On the other hand, Cubans, when compared to Italians reported higher scores for food thought suppression with reward responsiveness and restrained eating emerging as significant predictors of between-country differences. To conclude, eating behaviors in Cubans could be different from those reported in European countries, perhaps as a consequence of Cuba’s recent history.

## Introduction

Cubans have been living under rationing since the 1960’s, and generations of Cubans have grown-up experiencing numerous restrictions including food rationing. This is mainly attributed to the combination of both the US embargo, the longest in the modern history (Garfield and Santana, 1997), and internal inefficiencies of the centralized economy (Anderson, 2014).

Food rationing was most extreme during the so-called “special period” (1990–1995), which followed the dissolution of the Soviet bloc in 1989. During these years Cuba’s gross domestic product declined by 60%, causing a concurrent fall in the caloric intake of adults from 3100 kcal/day in 1989 to 1863 kcal/day in 1994 (Garfield and Santana, 1997). This drastic fall in energy intake has been considered as a semi starvation condition (Kalm and Semba, 2005).

Although the population’s obesity rates began to increase when the “special period” came to an end (Jiménez-Acosta et al., 2012), food rationing remains to this day. Thus rationing continues to limit the increase in food availability among Cubans. As a consequence of its recent history eating behaviors among Cuban adults could have changed in the last decades. For example, it has been suggested that the perception of food scarcity could promote overeating (Laran and Salerno, 2013), and may weaken the motivation to exert self-control in the individual (Hofmann and Kotabe, 2012). Hence, restrained eating and dieting in Cubans (Lowe et al., 2013) may be less likely to be reported with a similar prevalence as in other countries with wider food availability.

Congruent with this hypothesis, independent studies conducted in Cuba and Italy seem to suggest that Cubans might have more severe food craving than their Italian counterparts (Innamorati et al., 2014; Rodríguez-Martín and Molerio-Pérez, 2014), and that food thought suppression could be an important attribute of eating behaviors among Cuban adults (Rodríguez-Martín et al., 2015). It has been observed that restrained eaters are especially susceptible to the behavioral effects of thought suppression (Erskine and Georgiou, 2010), and they are more likely to report more severe food craving than unrestrained eaters (Rodríguez-Martín and Molerio-Pérez, 2014).

Bearing in mind that studies on eating behaviors associated with this unique situation in Cuba are scarce or absent, we have to speculate on the current situation of Cuban people referring mainly to research conducted in Western developed countries (Elsner, 2003; Spinella and Like, 2004; Herman and Polivy, 2010; French et al., 2012; Meule and Vögele, 2013; Sominsky and Spencer, 2014).

Studies in these countries, where people have free access to food have demonstrated a relationship between sensitivity to reward and Body Mass Index (BMI; Franken and Murris, 2005; Davis et al., 2007), an association probably mediated by an increase in hedonic food intake. However, in an environment where preferred foods are not always available (either for the majority of the time or in sufficient amounts), eating behaviors may be driven by the maintenance of homeostasis rather than their hedonic value.

Thus, this is the first study which aims to directly investigate eating behaviors in Cuban adults, comparing them with what occurs in a developed Western country, Italy. Cuba and Italy present a set of different ethnographic and socio-economic characteristics and previous research has shown between-country differences with regard to emotional experience and subjective well-being (Galati et al., 2005, 2006; Sotgiu et al., 2011). Furthermore, the study aimed to investigate whether between-country differences in reward and inhibitory processes related to BMI are present, and whether reward responsiveness can be used to predict between-country differences.

According to the above described elements, the current exploratory research set out to answer the following questions: Do Cubans adults have more food craving and food thought suppression than Italian adults? Do Cubans report restrained eating and dieting less frequently? Is reward responsiveness a useful predictor of between-country differences? It was hypothesized that: (i) Cubans would report lower levels of restrained eating as well as a lower number of dieters; (ii) Cubans would report higher levels of food craving and food thought suppression; and (iii) reward responsiveness would play a key role in the ability to predict between-country differences.

As overweight and obese individuals (when compared with normal weight individuals) may show differences in eating behaviors and related variables (Spinella and Like, 2004; Fabbricatore et al., 2011a; French et al., 2012; Rodríguez-Martín et al., 2015), and reward and inhibitory processes which are likely to be associated with weight gain and obesity (Volkow et al., 2011), this study focused solely on individuals in the normal weight range with no history of obesity (Ely et al., 2013).

Furthermore, older adults were also excluded, because in this population there are frequently changes in the amount and type of food and nutrients consumed, in addition to the way they think about food (Elsner, 2003). An individual was considered to be an older adult when he/she was aged 60 years old and over. However, it must be noted there is not a consensus on the age which defines an “elderly” person; 60 years old and over is the cut off used by the World Health Organization (WHO, 2016).

## Materials and Methods

### Participants

Sample characteristics are displayed in **Table 1**. Participants were 283 normal weight adults from Cuba (*n* = 158) and Italy (*n* = 125), aged between 18 and 60 years old (Mean = 34.93; *SD* = 12.00). Around 52% of the participants were females.

**Table 1:** Comparison of the variables between countries.

	Italia	Cuba		Statistics	
			
Continuous variables	Mean ±*SD*		*t* (281)	*p*	*d*
**Restrained eating**	**25.18 ± 9.37**	**18.79 ± 7.29**	**6.445**	**0.000**	**0.76**
External eating	26.00 ± 7.19	26.93 ± 7.07	1.081	0.281	0.13
Emotional eating	24.02 ± 10.52	21.22 ± 6.82	2.699	0.007	0.32
Food cravings trait	27.69 ± 10.71	28.79 ± 8.82	0.943	0.346	0.11
**Food thought suppression**	**20.66 ± 9.65**	**27.33 ± 9.49**	**5.828**	**0.000**	**0.70**
**Drive**	**11.80 ± 2.64**	**6.58 ± 2.81**	**15.905**	**0.000**	**1.91**
**Fun seeking**	**11.47 ± 2.54**	**7.67 ± 2.81**	**11.898**	**0.000**	**1.41**
**Reward responsiveness**	**16.77 ± 2.32**	**8.48 ± 2.94**	**25.749**	**0.000**	**3.10**
**Behavioral inhibition**	**20.04 ± 3.80**	**12.55 ± 3.67**	**16.758**	**0.000**	**2.01**
Binge eating	5.55 ± 5.29	4.22 ± 4.09	2.370	0.000	0.28
BMI	22.65 ± 1.84	22.46 ± 1.61	0.933	0.351	0.11
Age	35.20 ± 11.63	34.72 ± 12.32	0.338	0.736	0.04

**Binary variables**	**No/Yes**		**χ^2^(1)**	***p***	***φ***

Female gender	66/59	71/87	1.427	0.232	0.078
Married or in a stable relationship	91/34	93/65	5.365	0.021	0.145
Higher education	77/48	84/74	1.695	0.193	0.085
Family history of obesity	92/33	115/43	0.000	0.985	0.009
Currently on diet	90/32	143/15	12.632	0.000	0.222

*Bold values mean significant differences with relevant effect sizes (from medium to large and large, respectively).*

Exclusion criteria included subjects which declared they were pregnant, lactating, had a history of obesity or eating disorders, or were unable to complete the assessment for other reasons including denial of informed consent. Finally, Cubans must have been born and resident in Cuba in order to guarantee that they had grown-up under rationing.

### Assessment

Socio-demographic/anthropometric data: Participants were asked to provide age, gender, education level, marital status, current diet, family history of obesity, height, and current weight (used to determine BMI).

#### Dutch Eating Behavior Questionnaire (DEBQ)

The DEBQ has 33 items forming three separate scales (van Strien et al., 1986): emotional eating (13 items: e.g., “Do you have a desire to eat when you are irritated?”), external eating (10 items: e.g., “If food smells and looks good, do you eat more than usual?”), and restrained eating (10 items: e.g., “Do you try to eat less at mealtimes than you would like to eat?”). Items were rated using a 5-point Likert-type scale (from 1, never to 5, very often). The current study has used both the Spanish (Cebolla et al., 2014) and Italian versions (Dakanalis et al., 2013), respectively.

#### The Behavioral Inhibition System/Behavioral Activation System (BIS/BAS) Scale (Carver and White, 1994)

The BIS/BAS scale measures individual differences in the BAS (approach) and the BIS reactivity by the degree to which respondents endorse prototypical approach-and avoidance-related behaviors. Items are rated using a 4-point Likert-type scale (from 1, disagree strongly to 4, agree strongly). The scale was divided according to its original structure: a single 7-item scale designed to assess BIS features, and three scales, Reward Responsiveness (5 items), Drive (4 items), and Fun Seeking (4 items) that assess different aspects of BAS functioning. The current study has used both the Spanish (Barranco Jiménez et al., 2009) and Italian versions (Leone et al., 2002).

#### Food Thought Suppression Inventory (FTSI)

This 15-item inventory was created based on the White Bear Suppression Inventory (Wegner and Zanakos, 1994), as a measure of food thought suppression (Barnes et al., 2009; Barnes and White, 2010). Participants responded to questions such as, “There are foods that I try not to think about” on a 5-point Likert scale ranging from 1 (*strongly disagree*) to 5 (*strongly agree*). The current study has used both the Spanish (Rodríguez-Martín et al., 2012) and Italian versions (Cronbach’s alpha = 0.93).

#### Food Cravings Questionnaire-Trait-Reduced (FCQT-r)

The FCQT-r is composed of 15 items from the FCQ-T (Meule et al., 2014). The items belonged to five dimensions of the original FCQ-T (Cepeda-Benito et al., 2000): Control (items 2, 3, 25, 26, 29), Thoughts (items 6, 8,27, 32, 33), Intent (items 5,18), Emotions (items 20, 34), and Cues (item 36). The current study has used both the Spanish (Rodríguez-Martín and Molerio-Pérez, 2014) and Italian versions (Innamorati et al., 2014).

#### Binge Eating Scale

This is a 16-item questionnaire assessing binge eating severity as well as the feelings and thoughts associated with such behavior (Gormally et al., 1982). It assesses both behavioral and cognitive/emotional manifestations of binge eating using 16 groups of statements. When rating each item, the respondent has to choose one of three or four response statements of increasing severity for each question. Both the Spanish (Partida et al., 2006) and Italian versions (Di Bernardo et al., 1998; Imperatori et al., 2015), were used.

### Procedure

The study was approved and ethical approval was granted by the scientific council at the Central University “Marta Abreu” of Las Villas (Cuba) and by the review board of the Università Europea di Roma (Italy). The study was performed between November 2014 and May 2015. All participants were informed in person about the study procedures by psychology students (called surveyors), and signed an informed consent prior to assessment.

Surveyors were trained for the sample selection and assessment (16 h at the authors’ institution). Training included lectures about eating behaviors and food cravings, as well as practical sessions on data collection. Sample selection was carried out in each surveyor’s geographical area by inviting members of the surrounding community to participate through verbal announcements. Surveyors were asked to assess individuals who agreed to complete the assessment and fulfilled the inclusion criteria. The time required to complete the questionnaires never exceeded 30 min. All the participants volunteered to partake in the study and no financial compensation was offered in return.

### Data Analysis

The statistical package SPSS 20.0 was used for data analysis. Comparisons between groups were performed using Chi-square and *t*-tests. Effect sizes were calculated with: (1) Cohen’s d from *t*-test values using ViSta 7, where values of 0.2, 0.5, and 0.8 were defined as small, medium, and large effects, respectively (Ledesma et al., 2009); and (2) phi (Φ) from Chi-square, where values of 0.1, 0.3, and 0.5 were defined as small, medium and large effects, respectively (Rice, 2009). All statistical tests reported are two tailed and *p* values marked as ns refer to *p* > 0.05.

A CHAID (Chi-squared Automatic Interaction Detector) algorithm was also used with *Cubans* as the target variable. Classification trees allow researchers to efficiently identify interactions between predictors without the need to anticipate and specify these in advance (King and Resick, 2014). In addition, classification trees are a very flexible easy to understand tool, and allow for the detection of non-linear interactions (Richards et al., 2008).

Chi-squared Automatic Interaction Detector relies on the Chi-square test to determine the best next split at each step in a classification problem, if the test shows a significant difference, a new category is created, and the next two classes are evaluated. This process is continued until no further splits can be made along any of the branches. A model near to 80% of correct classifications may be considered suitable (Nisbet et al., 2007).

## Results

### Differences between Nationalities

According to **Table 1**, there were between-country differences, with large effect sizes for BIS/BAS scales (*d* > 1.4), where Italians showed higher scores than Cubans. This trend was also found for restrained eating, with an effect size from *medium* to *large* (*d* = 0.76). Accordingly, there were more current dieters among Italian participants than among Cubans, despite the fact the effect size was small (ϕ = 0.22). On the other hand, when compared to Italian participants Cubans showed higher scores on food thought suppression with an effect size of *medium* to *large* (*d* = 0.70).

### Classification Tree

**FIGURE 1:**
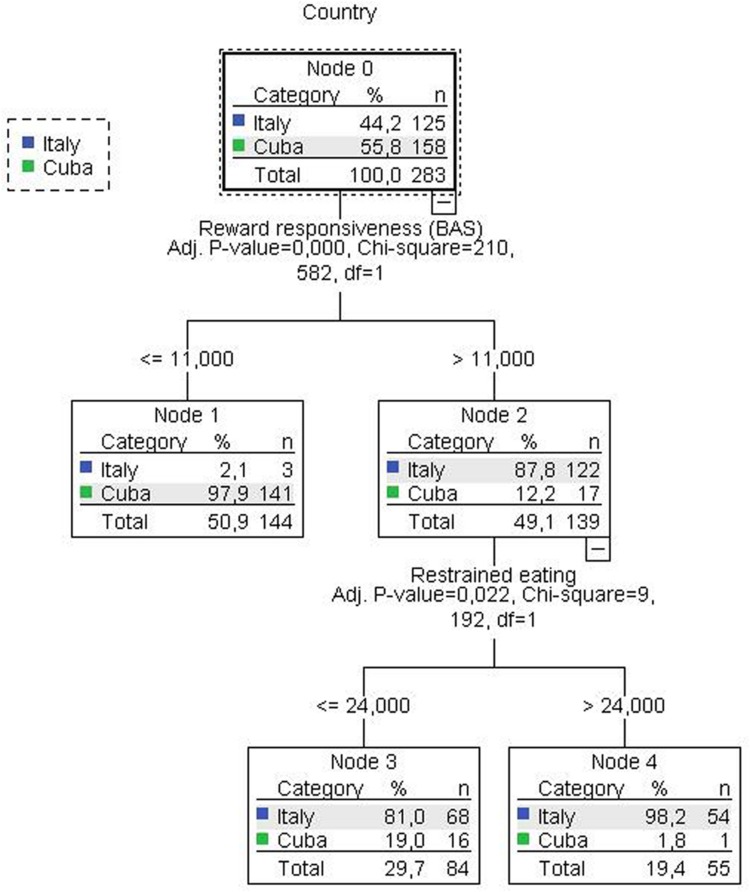
CHAID (Chi-squared Automatic Interaction Detector)-tree.

The obtained CHAID tree is shown in **Figure 1**. Country was introduced as a dependent variable and the remaining variables were introduced as predictors (**Table 1**). Only reward responsiveness and restrained eating emerged as significant predictors able to discriminate between countries. The model was adequate and the percentage of correct classification was above 90% for the whole sample (97.6% for Italians and 89.2% for Cubans).

The model obtained may be summarized as follows. The CHAID tree revealed that the participants’ country interacted first with reward responsiveness (Nodes 1 and 2). The majority of individuals with scores higher than 11 points were Italians (87.8%: Node 2), whereas the opposite was observed for lower scores (Node 1, Cubans: 97.9%). Finally, individuals grouped in Node 2 (Reward responsiveness score >11) were also differentiated by severity of restrained eating, where the proportion of Cubans decreased considerably among individuals with higher scores (<2%: Node 4).

## Discussion

Results suggest that normal weight Cuban and Italian adults may be differentiated between by their eating behaviors as well as their temperamental traits. Contrary to what was hypothesized from results of two independent studies (Innamorati et al., 2014; Rodríguez-Martín and Molerio-Pérez, 2014) no between-country difference was found for the food craving trait.

The observed between-country differences in the BIS/BAS scales add support to supposed variations in internal stimuli eliciting eating behaviors. Speculatively, it may be suggested that Cuban adults mainly eat in response to physiological hunger, inhibiting food intake once satiated; whereas Italians eat regularly for hedonic reasons. According to a recent review, elevated impulsivity and high delay discounting (i.e., a devaluation of a reward over time) may contribute to increased hedonic hunger (Ely et al., 2013). In Cuban adults, healthy weight individuals may have a better interoceptive sensitivity determining more functional signals of hunger and satiety (Herbert et al., 2013), possibly due to the lack of a systematic availability of highly palatable foods.

Results for this study concerning measurements which assess eating behaviors are consistent with the above findings. Sensitivity to reward has been reported to be directly related with measurements of overeating, such as restrained, emotional, and external eating, as well as preferences for sweet and fatty foods (Davis et al., 2007). It has also been observed that BIS reactivity could be positively related with binge eating (Fabbricatore et al., 2011b). The higher proportion of current dieters among Italian participants also supports this idea. Normal weight individuals who diet may do so because of their resistance to a powerful predisposition to hedonic eating and weight gain (Lowe et al., 2013).

The absence of significant between-group differences for food cravings was an unexpected result; however, it could be explained by the role of food thought suppression in controlling food cravings. According to the Elaborated Intrusion Theory of Desire (Kavanagh et al., 2005; May et al., 2012, 2014) the experience of craving is a cycle of cognitive elaboration of initial intrusive thoughts elicited by drug or food related stimuli. If the desire cannot be satisfied, the experience becomes unpleasant and people may try to suppress these thoughts. This strategy is counterproductive, exacerbating rather than reducing the occurrence of intrusive thoughts (Rodríguez-Martín et al., 2013), producing the elaborative cognitive processes cycle (May et al., 2014). It has also been observed that food thought suppression decreases activity in the core of the mesocorticolimbic circuitry (i.e., ventral tegmental area and ventral striatum), which can be interpreted as the inhibition of the behavioral expression of reward processing (Siep et al., 2012). Perhaps food thought suppression could be considered as an effective cognitive strategy in controlling food cravings among Cuban participants.

According to our predictive model, reward responsiveness was a key factor for the classification of participants from both countries. At the same time, higher scores on reward responsiveness were linked with restrained eating. The model could be interpreted as the main features of both food-rationing and food-availability on the appetitive system and eating behaviors among healthy weight individuals.

Food restrictions were imposed as a part of general rationing of goods among Cubans in their everyday life whereas this has not been a factor for Italians. Thus, the latter may have developed more need to control their reward responsiveness through behaviors such as restrained eating or dieting. It should be taken into account that dieters and restrained eaters do not differ in terms of an underlying tendency toward weight gain, despite the fact that restrained eating could represent a more effective means of preventing it (Lowe et al., 2013).

Although the results are interesting, their limitations should be considered. First, the investigation was an exploratory cross-sectional study based on self-reported assessment. Secondly, the sample was non-representative. Thirdly, both caloric intake and food accessibility were not controlled. Finally, a bias in cross-country surveys when comparing mean scores on psychometric measures should be taken into account. For example, for most of the questionnaires used there are not studies investigating the structural invariance of the Italian and Cuban versions of the measurements.

## Conclusion

Cuban adults (who have been living under food rationing for several years) reported a different temperamental profile and less restrained eating than their counterparts from Italy; whereas the opposite was found for food thought suppression. Reward responsiveness and restrained eating were the main predictors of the participants’ country. These results should encourage researchers to explore the effects of prolonged food-rationing on human eating behaviors in much greater depth. On the other hand, clinicians should take the observed between-country differences in the way that people think about food into account in order to provide better health care services.

## Author Contributions

BR-M and MI participated in the design of the study. BR-M and MI were also responsible for statistical analysis and for writing statistical sections of the manuscript. All authors (BR-M, MI, CI, MF, DH, LJ, and SR-S) contributed to critically revising the work, sample collection, approved the final version of the article to be published and agreed to be accountable for all aspects of the work in ensuring that questions related to the accuracy or integrity of any part of the work are appropriately investigated and resolved.

## Conflict of Interest Statement

The authors declare that the research was conducted in the absence of any commercial or financial relationships that could be construed as a potential conflict of interest.
